# Association of Digital Health Literacy with Future Anxiety as Mediated by Information Satisfaction and Fear of COVID-19: A Pathway Analysis among Taiwanese Students

**DOI:** 10.3390/ijerph192315617

**Published:** 2022-11-24

**Authors:** Sheng-Chih Chen, Le Duc Huy, Cheng-Yu Lin, Chih-Feng Lai, Nhi Thi Hong Nguyen, Nhi Y. Hoang, Thao T. P. Nguyen, Loan T. Dang, Nguyen L. T. Truong, Tan N. Phan, Tuyen Van Duong

**Affiliations:** 1 Graduate Program of Digital Content and Technologies, College of Communication, National Chengchi University, Taipei 116-05, Taiwan; 2 Health Personnel Training Institute, University of Medicine and Pharmacy, Hue University, Hue 491-20, Vietnam; 3 Department of Radio, Television & Film, Shih Hsin University, Taipei 116-42, Taiwan; 4 Department of Education, National Taichung University of Education, Taichung 403-06, Taiwan; 5 School of Health Care Administration, College of Management, Taipei Medical University, Taipei 110-31, Taiwan; 6 School of Nutrition and Health Sciences, Taipei Medical University, Taipei 110-31, Taiwan; 7 Institute for Community Health Research, University of Medicine and Pharmacy, Hue University, Hue 491-20, Vietnam; 8 Faculty of Nursing and Midwifery, Hanoi Medical University, Hanoi 115-20, Vietnam; 9 School of Nursing, National Taipei University of Nursing and Health Sciences, Taipei 112-19, Taiwan; 10 Pharmacy Department, School of Medicine, Vietnam National University, Ho Chi Minh City 700-00, Vietnam; 11 Pharmacy Department, Thong Nhat Hospital, Ho Chi Minh City 721-18, Vietnam; 12 Department of Tropical Diseases, Cho Ray Hospital, Ho Chi Minh City 727-13, Vietnam; 13 International Health Program, College of Medicine, National Yang Ming Chiao Tung University, Taipei 112-304, Taiwan; 14 International Master/Ph.D. Program in Medicine, College of Medicine, Taipei Medical University, Taipei 110-31, Taiwan

**Keywords:** digital health literacy, fear, COVID-19, social status, future anxiety, information satisfaction, pathway analysis, students, Taiwan

## Abstract

Digital Health Literacy (DHL) helps online users with navigating the infodemic and co-existing conspiracy beliefs to avoid mental distress and maintain well-being. We aimed to investigate the association between DHL and future anxiety (FA); and examine the potential mediation roles of information satisfaction and fear of COVID-19 (F-CoV). A web-based cross-sectional survey was carried out among 1631 Taiwanese university students aged 18 years and above from June 2021 to March 2022. Data collected were socio-demographic characteristics (sex, age, social status, university location), information satisfaction, F-CoV, DHL and FA (using Future Dark scale). The linear regression model was used to explore factors associated with FA. The pathway analysis was further used to evaluate the direct and indirect relationship between DHL and FA. A higher score of DHL (B = −0.21; 95% CI, −0.37, −0.06; *p* = 0.006), and information satisfaction (B = −0.16; 95% CI, −0.24, −0.08; *p* < 0.001) were associated with a lower FA score, whereas a higher F-CoV score was associated with a higher FA score (B = 0.43; 95% CI, 0.36, 0.50; *p* < 0.001). DHL showed the direct impact (B = −0.1; 95% CI, −0.17, −0.04; *p* = 0.002) and indirect impact on FA as mediated by information satisfaction (B = −0.04; 95% CI, −0.06, −0.01; *p* = 0.002) and F-CoV (B = −0.06, 95% CI, −0.08, −0.04; *p* < 0.001). Strategic approaches to promote DHL, information satisfaction, lower F-CoV are suggested to reduce FA among students.

## 1. Introduction

After the official reporting of the first case in Wu Han, China, in December 2019, there were over 626 million COVID-19 cases worldwide, with approximately 6.6 million COVID-19 deaths, as of 31 October 2022 [1]. In Taiwan, the COVID-19 pandemic was attributed to more than 7.7 million cases and nearly 13 thousand deaths by 31 October 2022 [2]. The highest COVID-19 incidence and mortality rates were mainly located in the northern part of Taiwan, particularly in Taipei city and New Taipei city [3]. In response of pandemic, Taiwanese government implemented a wide range of non-pharmaceutical interventions such as border control, home quarantine, and contact tracing [3]. Implementing these measures emphasized harmonization and flexibility to avoid hard lockdowns, which often caused restriction of civil freedom and negative economic impacts [3,4].

During the COVID-19 era with a variety of social restriction measures [5,6], a higher frequency of information-seeking behavior and a larger number of Internet users have been reported [7]. The Internet and social media are quickly becoming the primary source for individuals, especially among university students, to provide the most recent health messages and update public health policies [8,9,10]. Over 2020, the percentage of users on Facebook and TikTok grew by 8% and 38%, respectively [11].

Aside from the advantages of social media and the Internet, online users usually cope with a concern known as infodemic. The World Health Organization (WHO) defined the term “infodemic” as the overabundance of information on the Internet or other communication technologies, where spread both valid and invalid information during a disease outbreak [12]. Several studies indicated that university students found it challenging to identify reliable sources for COVID-19-related information. In Germany, Dadaczynski et al. found that 30.4% students reported difficulties in searching accurate information on specific health-related themes when assessing the reliability of Internet information [13]. Similarly, 49.3% of students in Slovenia had difficulty in accessing reliable web materials [14]. Previous studies showed that more exposure to inauthentic news and greater conspiracy belief were associated with some form of anxiety [15]. In 1996, Zaleski claims that certain forms of anxiety had a link with the anticipated future [16]. This kind of anxiety is expressed by the state of uncertainty, fear or worries that they believe it is more likely to happen to them [16].

During the COVID-19 period, the future anxiety (FA) related to career plans among university students might be worsen due to the uncertainty of global economies and industries [17,18,19]. International Monetary Fund predicted that the world would break the record of economic recession since the World War II [20] that led to a lower gross domestic product growth rate and a higher percentage of unemployed [21,22]. In addition, FA among university students might be attributed to maintaining academic performance when transiting to virtual learning [23,24]. Certain studies indicated contributors to stress during the online learning process including poor access to hands-on experiment work [25], lack of interactive communication, understanding, motivation [26], and loss the study focus [27]. Furthermore, prior studies showed that those with higher FA were less able to focus on solving problem practice and pursuing positive health behaviors [28,29], which leads to lower overall well-being [30].

Fear of COVID-19 (F-CoV) could also play an important role in causing people’s anxiety about the future. Beck and Emery suggested that fear is the perception and evaluation of risk, and anxiety is the unpleasant emotional state and physiological reaction that happens when fear is induced [31]. With access to real-time information during the COVID-19 pandemic, unverified information and increased social media rumors produce a new kind of fear called F-CoV [32]. The alert level of F-CoV has been reported in many countries, leading to healthcare access delays [33,34] and increasing suicide rates [35]. The coexisting of this fear and the uncertainty about the future might affect FA in the university population. A previous study at a Bangladesh university found that F-CoV is directly associated with FA [17]. Therefore, we hypothesized that there is a positive association between F-CoV and FA.

In contrast, information satisfaction is considered an important factor to improve mental well-being among students [36]. People having higher satisfaction with the information demand are more likely to better understand the COVID-19 situation and avoid the anxiety caused by the overwhelming misinformation [37]. In a study on Chinese students in China and South Korea during COVID-19, online information satisfaction was negatively associated with psychological problems [38]. We assumed that information satisfaction had a negative association with FA.

In addition, information satisfaction has been proven to have positive relationship with Digital Health Literacy (DHL) [38]. DHL serves as a critical factor in assisting online users in accessing, understanding, evaluating, and using information and services to maintain and promote health and well-being [39]. DHL has the similar objective as health literacy, which assesses the capacity to manage and apply health-related information to make health decisions; however, DHL focuses more on health information obtained via online platforms [39,40]. Recent reports have shown that having adequate DHL is an important and predisposing factor for navigating the “infodemic” and “co-existing conspiracy beliefs” [41,42], and adopting appropriate measures to alleviate negative thoughts about the future during the pandemic [43]. However, the proportion of students with adequate DHL remained at moderate level. Previous studies have reported prevalence rates of adequacy DHL among students in Germany, the United States and Pakistan were 49.9% [44], 49.0% [45] and 54.3% [9]. Meanwhile, university students often show higher digital capabilities and more frequency of online exposure than other groups [28]. A previous study indicated more frequency of social media exposure was significantly associated with a higher risk of anxiety and depression [46]. Especially, people with low DHL are more vulnerable to the F-CoV and more at risk of other psychological disorders, because they are less likely to navigate misleading information [47]. A recent study in Hong Kong found that DHL has a negative association with FA among the elderly [48]. However, the relationship in university population were not highlighted. Lack of study examine indirect effects and the role of mediators in the relationship between DHL and FA among the university students, for example F-CoV and information satisfaction.

Therefore, this study aimed to identify the association between DHL and FA and examine the potential mediation roles of information satisfaction and F-CoV in the relationship between DHL and FA. The researcher’s hypotheses were presented in Table 1.

## 2. Materials and Methods

### 2.1. Study Design and Participants

A web-based cross-sectional study was conducted from June 2021 to March 2022 among university students in Taiwan. The online survey (Supplementary Materials) was sent out to students via the university email system, or posted on social media platforms, e.g., Facebook and Line.

Students aged 18 years or older, studied at universities were invited to join the survey. A total of 1631 students completed the online questionnaire. Their data were coded and analyzed anonymously.

### 2.2. Ethical Consideration

The study was approved by the Research Ethics Committee of National Chengchi University (IRB No. NCCU-REC-202106-I066). Students participated voluntarily and signed the online informed consent form before taking the survey. They were informed about the purpose of the study and data usage. They can withdraw or stop answering the question at any time-point.

### 2.3. Instruments and Measurements

The Chinese questionnaire was used as a part of the COVID-HL Network Survey on University Students [9,28,30]. The information related to sociodemographic characteristics, subjective social status, information satisfaction, F-CoV, DHL, and FA were collected. The documentation on these measurements and their calculation was well described in a previous study [49].

#### 2.3.1. Sociodemographic Information

The sociodemographic part collected information on the sex (male/female), age, study program (bachelor/masters/other), the university discipline, the university location (north, center, south, east, and outlying islands). 

#### 2.3.2. Subjective Social Status

The subjective social status was evaluated using the MacArthur Scale [50]. The respondents were asked a question about their position on an eleven-point scale, which represents where people stand in Taiwan.

#### 2.3.3. Digital Health Literacy

DHL was assessed using the DHL Instrument (DHLI) developed by Van der Vaart (2017) [51]. This DHLI was adjusted to the COVID-19 pandemic and focuses on (1) information seeking, (2) contributing self-generated content, (3) assessing reliability, (4) deciding relevance, and (5) preserving and respecting privacy. Participants were asked to select a score on a four-point Likert scale. Since the privacy domain of DHLI had low reliability score (α = 0.55), we excluded the privacy related items in calculating overall DHL score. The DHLI reached a good level of reliability (α = 0.88) [52]. In this research, the DHL was considered as the most important independent variable associated with FA.

#### 2.3.4. Information Satisfaction 

The students were asked to indicate their satisfaction with the information they found on the Internet about the coronavirus. The question was recorded on a five-point scale from 1 (very dissatisfied) to 5 (very satisfied). The information was considered as an independent variable and a potential mediator of association between the DHL and FA.

#### 2.3.5. Fear of COVID-19 

F-CoV was evaluated using the 7 items in the F-CoV scale developed by Ahorsu et al. (2020) [32]. The F-CoV was used as an independent variable and mediators of association between the DHL and FA in this study. The F-CoV has a Cronbach alpha of 0.88 which indicated a good reliability scale [52].

#### 2.3.6. Future Anxiety

We adopted the five-item future dark scale to measure FA, which is the key dependent variable in our study. Each item was recorded with a seven-point Likert scale [16]. In this study, the FA scale reached good reliability (Alpha = 0.88) [52].

### 2.4. Data Analysis

Data were analyzed using R (Version 4.3.1) and RStudio. We carried out the descriptive analysis to present the mean, standard deviation, frequency, and percentage of the sociodemographic characteristics as dependent and independent variables. The overall scores of each scale, including FA, F-CoV, and DHL were calculated. 

We utilized a multivariable linear regression analysis to explore the significant factors associated with FA. The variance inflation factor was evaluated to check the multicollinearity between the independent variables. A *p*-value of below 0.05 was considered statistically significant.

**Figure 1:**
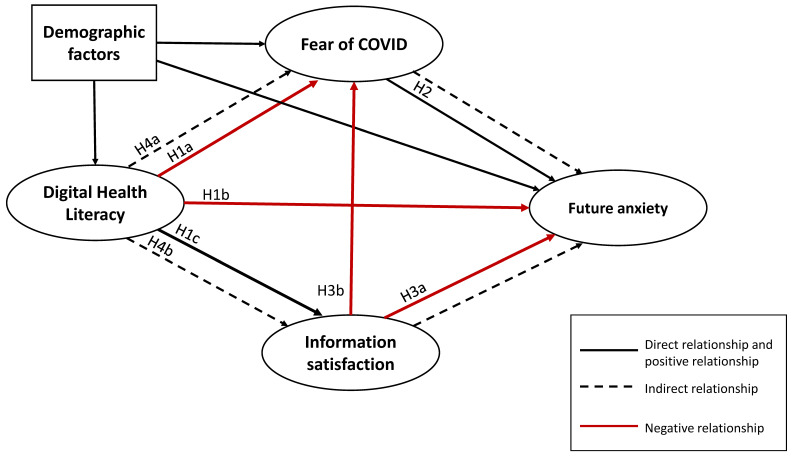
The conceptual framework.

Based on the literature review, we created a conceptual framework including the relationship between DHL and information satisfaction, F-CoV and FA (Figure 1) [16,17,18,19,30,31,32,38,46,48,53]. The structural equation model (SEM) was used to analyze the indirect effects, direct effects, and total effects of the DHL on FA. The SEM consists of a confirmatory measurement model, sometimes referred to as the confirmation factor analysis (CFA), which estimates the relationships between latent constructs and their manifested variables, and a structural model, which calculates the relationships between constructs [54]. The SEM demonstrated advantages over regression analysis in terms of (1) the modeling of measurement errors and unexplained variances, (2) the simultaneous testing of correlations, (3) the ability to combine micro- and macro-perspectives, and (4) the formulation of the most appropriate model and theory [55]. We utilized the lavaan package in R to establish the SEM and conduct the pathway analysis [56].

## 3. Results

Out of studied participants, 73.1% were female, 49.0% attended universities in northern Taiwan, 75.3% pursued a bachelor’s degree, and 90% enrolled in non-health-related programs. The mean age, mean scores of DHL, F-CoV, information satisfaction, subjective social status, FA were 22.6 ± 4.7, 3.1 ± 0.4, 2.5 ± 0.8, 3.5 ± 0.7, 6.0 ± 1.4, 4.4 ± 1.2, respectively (Table 2).

Respondents studying a bachelor’s degree and in northern universities had higher score of FA than other groups (*p* < 0.001). Furthermore, correlation coefficients showed that FA was positively correlated with the F-CoV and negatively correlated with age, DHL, information satisfaction, and subjective social status (Table 2).

**Figure 2:**
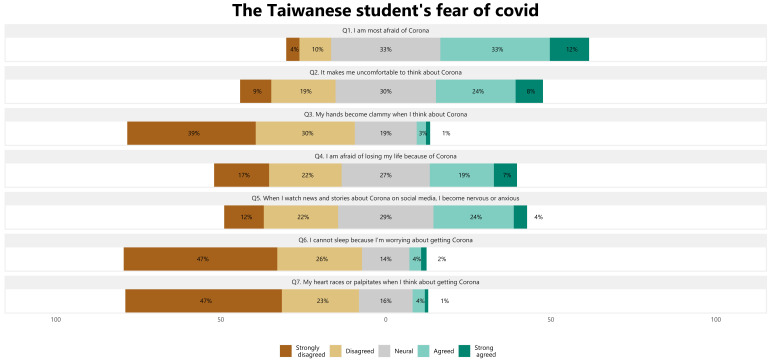
The distribution of the F-CoV questions among Taiwanese students.

Figure 2 shows the percentages of student responses for each question about the F-CoV. Among university students who answered the F-CoV subscale, 33% agreed and 12% strongly agreed that Coronavirus was their most feared. Besides, the number of participants was nearly balanced between agreeing and disagreeing on the questions about mental health related to COVID-19, such as feeling uncomfortable thinking about Coronavirus (24% agreed, 19% disagreed), afraid of losing their life because of Coronavirus (19% agreed, 22% disagreed), and become nervous or anxious when watching the news about Coronavirus (24% agreed, 22% disagreed). However, the percentage of participants with mental-related physical conditions was low. In particular, when thinking or worrying about Coronavirus, 3% of respondents agreed that their hands became clammy, 4% of respondents could not sleep, and 4% of them experienced racing heartbeat.

**Figure 3:**
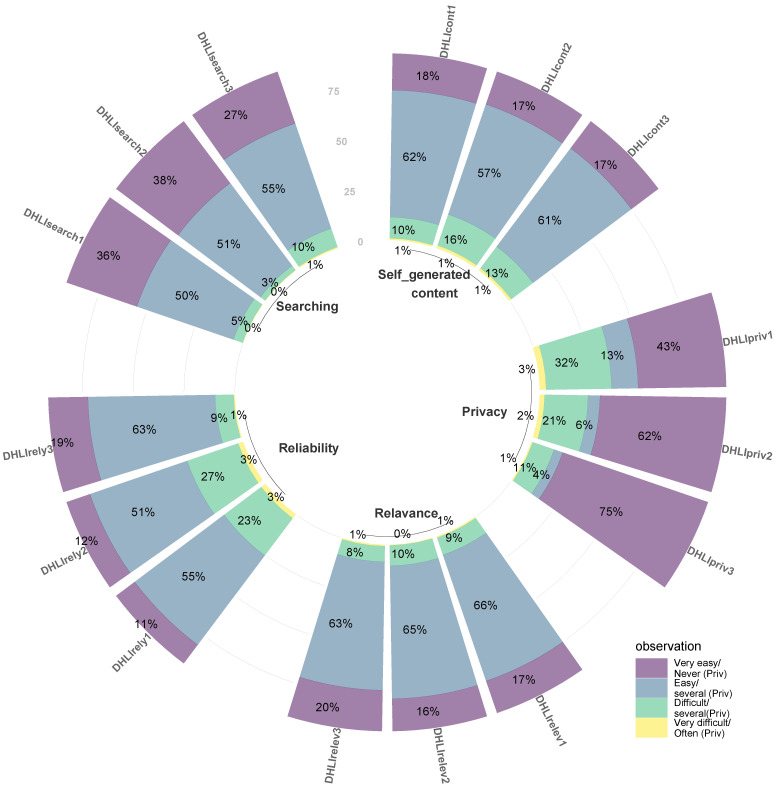
The distribution of digital health literacy (DHL) questions among Taiwanese students. Abbreviations: DHLIsearch1—Make a choice from all the found information; DHLIsearch2—Use the proper words or search query to find the information; DHLIsearch3—Find the exact information they are looking for; DHLIcont1—Clearly formulate question or health related inquiries; DHLIcont2—Express your opinion, thoughts, or feelings in writing; DHLIcont3—Write your message as such, for people to understand exactly what they means; DHLIrely1—Decide whether the information is reliable or not; DHLIrely2—Decide whether the information is written with commercial interests; DHLIrely3—Check different websites to see whether they provide the same information; DHLIrelev1—Decide if the information they found is applicable to them; DHLIrelev2—Apply the information them found in their daily life; DHLIrelev3—Use the information they found to make health related decisions; DHLIpriv1—Judge who can read their messages; DHLIpriv2—Share their own private information (e.g., name or address); DHLIpriv3—Share some else’s private information.

Figure 3 demonstrates the distribution of student’s responses about sub-domains of DHL. Regarding the “information search” subscale, the majority of university students reported that making a choice from all the information (86.0%), using proper words and search queries (89.0%), and finding exact information (82.0%) were easy and very easy. In terms of the dimension of “adding self-generated content”, respondents reported the most difficulties in expressing their own opinion, expressing thoughts or feelings in writing (16%), and in writing an understandable message (13%). Through all categories, the most significant difficulties could be found in the “protecting privacy” and “reliability” subscales. Around 35% of university students experienced difficult and very difficult in judging who could read messages posted on the web, while 15% of respondents said they sometimes or often shared private information on the website. For reliability, the highest proportion of university students found it challenging to determine whether the information was written with commercial interest (30%) and whether it was reliable (26%).

**Figure 4:**
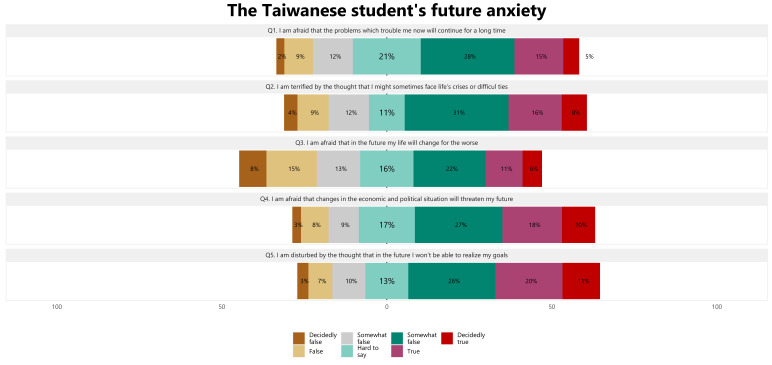
The distribution of FA questions among the Taiwanese university students.

Figure 4 presents the percentage of students’ responses to each question on the FA. Across all dimensions, we found that the largest proportions of respondents reporting true or definitely true could be found in the items related to being troubled by the idea that they will not be able to achieve their goals in the future (31.0%) and fearing that unwanted economic and political situation will negatively affect their future (28.0%). Nearly 20% of university students reported being fearful of difficulties that affect them today and would persist for a long time. In comparison, 4.0% of students were horrified by the idea that they sometimes experience life’s crises or tough relationships. The statement “I fear that my life may change for the worse in the future” gained agreement from 17.0% of respondents.

### 3.1. Associated Factors of Future Anxiety

We next explored the relationships between FA and factors among university students in Taiwan. Table 3 showed that FA score was positively associated with the F-CoV and negatively associated with age, higher education, center university location, DHL, information satisfaction, and subjective social status. Besides, each variable explains a small proportion of the variance of FA, with F-CoV explaining the highest amount (R^2^ = 8.0%), followed by subjective social status (R^2^ = 5.6%), and DHL (R^2^ = 1.9%). 

A multiple regression analysis reported an R^2^ of 16.1% when all the associated variables were included. Participants studying at the universities in central Taiwan had significantly lower FA scores than those studying in northern area (B = −0.20; 95% CI, −0.33, −0.07; *p* = 0.003). Besides, a higher FA score was negatively associated with a higher score of subjective social status (B = −0.19; 95% CI, −0.23, −0.15; *p* < 0.001), DHL (B = −0.21; 95% CI, −0.37, −0.06; *p* = 0.006), and information satisfaction (B = −0.16; 95% CI, −0.24, −0.08; *p* < 0.001). By contrast, students’ F-CoV were significant predictors of FA (B = 0.43; 95% CI, 0.36, 0.5; *p* < 0.001; Table 3).

### 3.2. Mediating Impacts of Information Satisfaction and Fear of COVID-19

In order to examine the relationship between variables and estimate the direct and indirect effects of DHL on FA, we carried out the pathways analysis. Pathway analysis has a distinct advantage compared to other conventional regressions, such as analyzing multiple regressions simultaneously and examining correlated measurement errors [57]. Therefore, it can reduce measurement errors, leading to better estimate results than traditional regression analysis [57,58].

**Figure 5:**
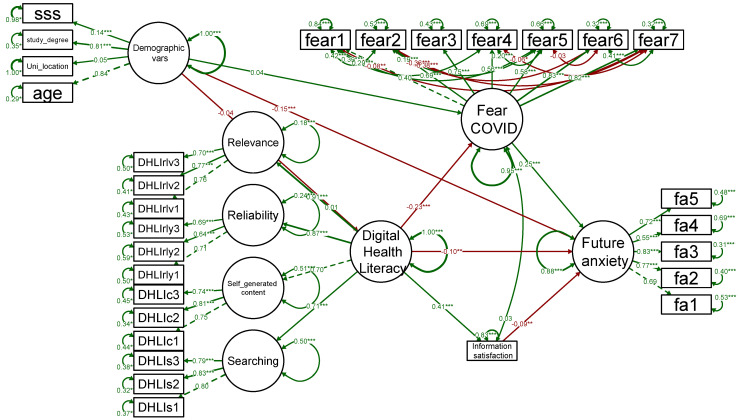
Pathway analysis for digital health literacy, future anxiety, and mediators. Abbreviation: fa—Future anxiety, fear—Fear of COVID-19, DHLIrlv—Subscales of DHL in relevance, DHLIrly—Subscales of DHL in reliability, DHLIc—Subscales of DHL in self-generated contents, DHLIs—Subscales of DHL in searching information, * for *p* less than 0.05, ** for *p* less than 0.01, and *** for *p* less than 0.001, red color text for negative coefficients, green color text for positive coefficients.

Figure 5 shows our final result of pathway analysis on the relationship among F-CoV, information satisfaction, DHL, and FA. The model has good model fit parameters with CFI = 0.95; NFI = 0.94; TLI = 0.95; GFI = 0.95; AGFI = 0.9, SRMR = 0.041; RMSEA = 0.039; CRMR = 0.043. The model indicated that DHL (B = −0.104, *p* = 0.002) and information satisfaction (B = −0.091, *p* = 0.002) were negatively associated with FA. In contrast, the F-CoV (B = 0.254, *p* < 0.001) was positively linked with the level of FA. We also found that the higher level of DHL was significantly associated with greater information satisfaction (B = 0.414, *p* < 0.001), and lower level of the F-CoV (B = 0.225, *p* < 0.001).

Table 4 shows the results of the mediation analysis by using the SEM. F-CoV and information satisfaction were two mediators which explained 20% and 30% the association between DHL and FA, respectively. Digital health literacy was negatively associated with FA score in the total effects (B = −0.20; 95% CI, −0.25, −0.14; *p* < 0.001). Besides, the standardized regression coefficients for the association of DHL and FA mediated by information satisfaction and F-CoV were −0.04 (95% CI, −0.06, −0.01; *p* = 0.002) and −0.06 (95% CI, −0.08, −0.04; *p* < 0.001), respectively.

## 4. Discussion

In our study, FA was positively associated with the level of DHL and information satisfaction, but negatively associated with the F-CoV. Furthermore, after conducting pathway analysis, DHL showed the direct and indirect impact on FA as mediated by information satisfaction and F-CoV among Taiwanese students during the COVID-19 pandemic. Our results also indicated that the Taiwanese university students have a relatively good score of DHL.

To our knowledge, this is the first research to investigate the whole direct and indirect relationship between DHL and FA among university students during a pandemic. Few studies have evaluated the influence of the COVID-19 pandemic on FA among university students; however, the role of DHL was not highlighted. Several research examined the relationship between future worry and feeling of coherence in elderly people [48] and the F-CoV among Bangladesh university students [17]. Another research in the United Kingdom measured the difference in FA and DHL between non-health care and health care students, yet it did not investigate the link between these two variables [28].

In 1996, Zaleski proposed the term of FA and claimed that many types of worry had a connection to the expected future [16]. The increased future concern may be attributable to health-related concerns, prospective economic and political uncertainty [16,29]. Additionally, a growing evidence revealed that the infodemic raise the public anxiety and psychological wellbeing during COVID-19 pandemic [59,60,61,62].

Our regression model found that DHL, information satisfaction, and the F-CoV were significantly associated with FA. In addition, prior research reported higher levels of DHL were linked with lower levels of the F-CoV [61] and more information satisfaction [37]. Therefore, we hypothesized that the association between DHL and FA might be mediated by the F-CoV and information satisfaction. Our results of pathway analysis indicated that with the presence of the F-CoV and information satisfaction, played a role as mediators in the indirect relationship between DHL and FA had significant and resulting in a full mediation. Our finding was in contrast with the previous result, which noted that there was no significant relationship between the eHEALs and FA [29]. However, the study was carried out on the adult population and did not examine the entire indirect association between DHL and FA. Hence, our study seems to provide further insight into the impact of COVID-19 on the mental health of student population.

As a surge of COVID-19 related to misinformation, advancing DHL plays a vital role in psychological distress, particularly in FA and F-CoV. In this study, the mean score of DHL was 3.1 ± 0.4, which was higher than the previous studies conducted in Vietnam (2.9 ± 0.3) [10], South Korea (2.9 ± 0.5) [63], and the United States (3.0 ± 0.5) [45]. When evaluating aspects of DHL, nearly 30% of student faces difficulties related to information reliability. Similar findings were observed in the prior studies conducted in Germany and Slovenia [14,64]. Dadaczynski et al. found that over 40% of German university students experienced difficulty determining the credibility of Internet information [64], whereas the figure for Slovenian students was 49.3% [14]. According to a research conducted in Japan, people who use a variety of information sources, including digital media and face-to-face contact, enriched more knowledge in terms of Health literacy and COVID-19 [65]. The other research indicated that students searching on public institution websites and health portal [13] had greater digital health capacities than those relying only on social media [66]. However, social media have been reported as the primary information source for the young population [65]. Hence, the government and authorities might consider implementing effective risk communication strategies on social media platforms to increase the number of valid information sources and limit misleading information. The goal could be achieved by working closely with social media companies or implementing a wide range of policies that control misinformation and avoid its consequences. During the COVID-19 pandemic, the WHO team aggressively engaged with digital giants such as Facebook, Twitter, and Tencent to prevent the spread of disinformation [67]. As a result, the student could navigate the online information more effectively and achieve better well-being.

In addition, our findings indicated that people with a low subjective social status and a younger age were likely to be more anxious about the future than other groups. Our result was consistent with previous research suggesting that people with a lower subjective social status were more prone to mental and physical health issues [68,69]. The university could establish policies or programs to assist these vulnerable populations when they encounter difficult living conditions, particularly during the COVID-19 pandemic. Our research also revealed that university students in northern Taiwan had a greater level of FA than counterparts in other regions. Currently, few studies compare the FA of the university population among different regions in Taiwan. In addition, our findings might not be representative of the Taiwanese university and mainly focus on the student who has carried out health information-related searches on the Internet. Therefore, our findings might pose suggestions for further studies in the future to confirm.

We admitted some limitations in our study. First, since this is the cross-sectional study, it is difficult to establish a causal association between the causes and FA. Moreover, we are unable to keep track of DHL or FA which can be changed with time-varying conditions of the COVID-19 pandemic. Second, our sample, which was collected via an online survey, is not representative of Taiwanese students as a whole. Therefore, the generalization of the results might be restricted. Lastly, our research did not include individuals who have no experience in online searching, which might have resulted in a selection bias.

## 5. Conclusions

The COVID-19 pandemic is not only posing health-related threats, but it also raises concerns about the health information crisis or called infodemic. In the present study, Taiwanese university students showed a relatively good score of DHL. However, more than 30% of them face challenges in evaluating information reliability. Therefore, developing critical thinking through training, workshop, and courses among university students play an important role in improving the ability to evaluate information and facilitate access to trustworthy information sources given by government portals and official health website related to COVID-19.

Importantly, this study provides the evidence regarding the relationship between DHL and FA among students during the COVID-19 pandemic. We used the pathway analysis and found that higher DHL expressed both direct and indirect associations with lower FA, while the information satisfaction and F-CoV indicated their mediating impacts on the association. In the future, it is necessary to develop and evaluate the effects of strategic approaches to promote DHL, information satisfaction, and lower F-CoV on FA in the university students. In addition, qualitative or mixed-method studies would be useful to explore how DHL influences FA in students.

## Figures and Tables

**Table 1:** List of research hypotheses.

	Types of Relationship	Hypotheses
H_1a,1b,1c_	Direct relationship	DHL is negatively associated with FA and F-CoV but positively associated with information satisfaction.
H_2_	Direct relationship	F-CoV is positively associated with FA.
H_3a,3b_	Direct relationship	Information satisfaction is negatively associated with FA and F-CoV.
H_4a,4b_	Indirect relationship	DHL has a significant relationship with FA mediated by the F-CoV and information satisfaction.

**Table 2:** Participants’ characteristics and future anxiety (*n* = 1631).

Factors	*n* (%)	FA Score	
		Mean ± SD	*p* Value *
Gender			0.920
Female	1193 (73.1)	4.4 ± 1.2	
Male	438 (26.9)	4.4 ± 1.3	
University location			0.001
North	800 (49.0)	4.5 ± 1.2	
Center	451 (27.7)	4.2 ± 1.3	
South	288 (17.7)	4.4 ± 1.2	
East and Outlying islands	92 (5.6)	4.4 ± 1.3	
Study degree			<0.001
Bachelor	1228 (75.3)	4.5 ± 1.2	
Master	373 (22.9)	4.2 ± 1.3	
PhD	30 (1.8)	4.3 ± 1.1	
Study program **			0.099
Health related program	158 (9.7)	4.3 ± 1.2	
Non-health related program	1473 (90.3)	4.4 ± 1.2	
Age, mean ± SD, *rho*	22.6 ± 4.7	−0.097	
DHL, mean ± SD, *rho*	3.1 ± 0.4	−0.113	
F-CoV, mean ± SD, *rho*	2.5 ± 0.8	0.283	
Information satisfaction, mean ± SD, *rho*	3.5 ± 0.7	−0.141	
Subjective social status, mean ± SD, *rho*	6.0 ± 1.4	−0.238	
FA, mean ± SD	4.4 ± 1.2		

Abbreviations: COVID-19, Coronavirus disease 2019; FA, Future anxiety; SD, standard deviation; *rho*, Correlation coefficient calculated by the Pearson test; * *p*-values based on the ANOVA test for differences of FA score in demographic factors. ** Health related programs including Medicine, Biomedical sciences, Pharmacy, Nursing, Medical Technology; Non-health related programs including Engineering Sciences/ICT, Linguistics & cultural studies/communication arts, Mathematics/natural sciences, Law and economics/criminology/public administration/political science, Social sciences/Social work/Psychology/Education, Business/Commerce/Accountancy/Management, Tourism/Hospitality/Hotel management, Architecture/design/arts/visual studies, Secondary level/Others).

**Table 3:** Associated factors of future anxiety via linear regression models (*n* = 1631).

Factors	Bivariate Model			Multivariate Model		
	B (95% CI)	*p*	R^2^	B (95% CI)	*p*	R^2^
Age	−0.03 (−0.04, −0.01)	<0.001	0.009	−0.01 (−0.03, 0.01)	0.189	0.161
Study degree			0.008			
Bachelor	Reference			Reference		
Master	−0.28 (−0.42, −0.14)	<0.001		−0.12 (−0.30, 0.06)	0.186	
PhD	−0.25 (−0.69, 0.20)	0.274		−0.1 (−0.53, 0.34)	0.660	
University location			0.008			
North	Reference			Reference		
Center	−0.29 (−0.43, −0.15)	<0.001		−0.2 (−0.33, −0.07)	0.003	
South	−0.10 (−0.27, 0.06)	0.230		−0.03 (−0.18, 0.13)	0.741	
East and Outlying islands	−0.12 (−0.38, 0.14)	0.377		−0.22 (−0.46, 0.03)	0.082	
Subjective social status	−0.21 (−0.25, −0.17)	<0.001	0.056	−0.19 (−0.23, −0.15)	<0.001	
DHL	−0.50 (−0.64, −0.35)	<0.001	0.025	−0.21 (−0.37, −0.06)	0.006	
Information satisfaction	−0.24 (−0.32, −0.16)	<0.001	0.019	−0.16 (−0.24, −0.08)	<0.001	
F-CoV	0.45 (0.38, 0.53)	<0.001	0.080	0.43 (0.36, 0.50)	<0.001	

Abbreviations: B, regression coefficient; CI, confidence interval; R^2^, variance; DHL, digital health literacy; COVID-19, coronavirus disease 2019.

**Table 4:** Mediating impacts of information satisfaction and F-CoV on the association between DHL and FA.

DHL	FA					
	Indirect		Direct		Total Effects	
	B (95% CI)	*p*	B (95% CI)	*p*	B (95% CI)	*p*
Information satisfaction	−0.04 (−0.06, −0.01)	0.002	−0.10 (−0.17, −0.04)	0.002	−0.20 (−0.25, −0.14)	<0.001
Fear of COVID-19	−0.06 (−0.08, −0.04)	<0.001				

Abbreviations: B, regression coefficient; FA, Future anxiety; DHL, digital health literacy; COVID-19, Coronavirus disease 2019.

## Data Availability

The data presented in this study are available on request from the corresponding author. The data are not publicly available due to ethical restrictions to protect the confidentiality of the participants.

## Supplementary Materials

The following supporting information can be downloaded at: https://www.mdpi.com/article/10.3390/ijerph192315617/s1, COVID-19 Health Literacy Survey: University Students (COVID-HL Survey).
