# Supplementary material for: Association of Digital Health Literacy with Future Anxiety as Mediated by Information Satisfaction and Fear of COVID-19: A Pathway Analysis among Taiwanese Students
Source: Int J Environ Res Public Health. 2022 Nov 24;19(23):15617. doi: 10.3390/ijerph192315617 (PMC9736687; doi:10.3390/ijerph192315617)
Supplement: Supplementary file 1 [file ijerph-19-15617-s001.zip › ijerph-1985477-supplementary.pdf]

COVID-19 Health Literacy Survey: University Students (COVID-HL Survey)

敬愛的同學您好：
針對COVID-19大流行所造成的許多不確定因素和不斷變化的社會環境，COVID-HL（COVID-19 and Health Literacy）聯合會正針對全球「大學生」開展一項關於COVID-19的（數位）健康識能調查。本研究「因應COVID-19大流行的數位健康識能調查：臺灣篇」為目前50多個參與調查國家中，以臺灣作為主要調查對象的國家；問卷採匿名且自願的方式進行填答，填答時間約20分鐘，您所提供的資料，將能使研究、政策和健康實踐在臺灣制定出更好的政策與方案，促進健康和保護行為、預防措施和防治COVID-19的政策，並能有機會更好地控制COVID-19及其傳播，為臺灣帶來更好的健康結果。
所有您提供資訊僅供學術研究使用，分析後將以統整方式進行結果呈現，不會呈現個人資料，敬請放心填寫。本研究由於為匿名填寫，無法提供中途退出研究之處理程序，但您在填答過程中感覺有疑問或敏感性問題，您可以隨時停止填答。
如果您於現在或填答期間有任何問題，請不必客氣，可與本計畫臺灣五位負責的教授聯繫；聯繫方式如下。您的意見對我們非常重要，衷心期盼您的回饋。最後感謝您的支持與協助！
敬祝健康平安！
計畫主持群：林承宇、賴志峰、陳聖智、Duong、黃詠愷 敬上

（聯繫資料）
計畫總主持人（PI, Taiwan）：林承宇／世新大學廣播電視電影學系所教授／電話：02-22368225轉83219／E-mail： cyou.lin@msa.hinet.net
主持人：賴志峰／國立臺中教育大學教育學系所教授／電話：04-22183052／E-mail：cflai@mail.ntcu.edu.tw
主持人：陳聖智／政治大學數位內容碩士學位學程副教授／手機：02-29393091轉66366／E-mail：scchen222@gmail.com
主持人：Duong, T. V.／臺北醫學大學保健營養學系助理教授／電話：02-27361661轉6545／E-mail：tvduong@tmu.edu.tw
主持人：黃詠愷／高雄醫學大學口腔衛生學系副教授／電話：07-3121101轉2209#63／E-mail：ykhuang@kmu.edu.tw

Dear students,
In response to the many uncertainties and changing social environment caused by the COVID-19 pandemic, the COVID-19 and Health Literacy (COVID-19) Consortium is conducting a (digital) study on COVID-19 for "university students" around the world. In this study, "Digital Health Literacy Survey in Response to the COVID-19 Pandemic: Taiwan Chapter", among the more than 50 participating countries, Taiwan is the main subject of the survey. The questionnaire was answered anonymously and voluntarily. The time to answer the questionnaire is about 20 minutes. The information you provide will support research, policy, and health practice in Taiwan in developing better policies and programs, promoting health and protective behaviors, preventive measures, and policies to combat COVID-19, and creating an opportunity to better control COVID-19 and its spread, ultimately leading to better health outcomes for Taiwan.
All of the information you provide is solely for academic research purposes. The findings will be presented in an unified way, with no personal information included. Please fill in your information with confidence. Due to the fact that this survey is done anonymously, there is no possibility to withdraw from the study in the middle of it. However, if you have any queries or encounter any sensitive situations while filling out the form, you can stop at any time-point.
If you have any questions during the answering, please contact the five academics in charge of this program in Taiwan; contact information is provided below. Your opinion is very important to us and we

sincerely look forward to your feedback. Finally, thank you for your assistance and support!
I wish you good health and safety!

Project host group: Lin Chengyu, Lai Zhifeng, Chen Shengzhi, Duong, Huang Yongkai Sincerely
(contact details)

Project host (PI, Taiwan): Lin Cheng Yu / Professor, Department of Radio, Television and Film, Shih Hsin University / Tel: 02-22368225 ext. 83219 / E-mail: cyou.lin@msa.hinet.net
Moderator: Lai Zhifeng / Professor, Department of Education, National Taichung University of Education / Tel: 04-22183052 / E-mail: cflai@mail.ntcu.edu.tw
Moderator: Chen Shengzhi / Associate Professor Cheng Cheng, Master of Digital Content, National Chengchi University / Mobile: 02-29393091 ext. 66366 / E-mail: scchen222@gmail.com
Moderator: Duong, T. V. / Assistant Professor, Department of Health and Nutrition, Taipei Medical University / Tel: 02-27361661 ext. 6545 / E-mail: tvduong@tmu.edu.tw
Moderator: Huang Yongkai / Associate Professor, Department of Oral Hygiene, Kaohsiung Medical University / Tel: 07-3121101 ext. 2209#63 / E-mail: ykhuang@kmu.edu.tw

大學生的問卷調查第一部分個人資料
Questionnaire for University Students - Personal Information

01. 填寫問卷前，我了解我的權益 Before filling out the questionnaire, I understand my rights

・我已閱讀問卷說明，並了解我的權益
I have read the questionnaire instructions and understand my rights
・否，我不同意（請勿繼續作答）
No, I disagree (do not continue answering)

02. 請問您的身份是？ Which group do you belong to?

・ 我是大學生，我想參加這次的調查。
I am a university student and would like to participate in the survey.

・ 我不是大學生，但我想參加這次調查。
I am not a university student, but I am interested in this survey.

03. 請問您的性別是？ Please indicate your sex.

・ 女性 Female ・ 男性 Male ・ 多元性別 Diverse

04. 請問您幾歲？ How old are you?

我_______歲。 I am______years old.

05. 您所就讀的系所？Please indicate the subject group of your course of study.

・ 工學院（工程科學、電機、機械工程等）Engineering Sciences
・ 文學院（語言、或文化研究等）Linguistics and Cultural Studies
・ 理學院（數學、自然科學等）Mathematics/Natural Sciences

・ 醫學院（醫學、藥學、公衛等）Medicine/Health Sciences
・ 法學院與商學院（法律、經濟等）Law and Economics
・ 社會科學院（社會、管理、心理、教育、傳播等）
Social Sciences/Social Work/Psychology/Education
・ 其他。請填寫___________________________
Other, namely ___________________________

06. 您就讀哪所大學？（請註明您的大學名稱）
What university are you studying? (Please indicate the name of your university.)

____

07. 請註明您的學校所在區域。**Please indicate the area where your university is located**

・ 北區（臺北市、新北市、桃園市、新竹縣、新竹市、宜蘭縣、基隆市）
North (Taipei City, New Taipei City, Taoyuan City, Hsinchu County, Hsinchu City, Yilan County, Keelung City)
・ 中區（苗栗縣、臺中市、彰化縣、南投縣、雲林縣）
Center (Miaoli County, Taichung City, Changhua County, Nantou County, Yunlin County)
・ 南區（臺南市、高雄市、屏東縣、嘉義縣、嘉義市）
South (Tainan City, Kaohsiung City, Pingtung County, Chiayi County, Chiayi City)
・ 東區（臺東縣、花蓮縣）
East (Taitung County, Hualien County)
・ 離島（澎湖縣、金馬特別行政區）
Outlying islands (Penghu County, Jinma Special Administrative Region)

08. 您是讀學士還是碩士課程？ **Are you studying in a Bachelor's or Master's programme?**

・ 學士 Bachelor　・ 碩士 Master　・ 其他 (例如：博士) Other (e.g. PhD)

09. 到目前爲止，您在大學裡已經入學了幾個學期（包括本學期）？
How many semesters (including the current semester) have you been enrolled at a university in so far?

__________學期 _______________semesters

10. 請想像一個梯子，代表臺灣人的分佈狀況。
Please think of a ladder as representing where people stand in TAIWAN.

說明：
❖ 排在上面：是那些最富有的人—那些錢最多、教育程度最高、工作最受尊敬的人等。
At the top of the ladder are the people who are the best off – those who have the most money, the most education, and the most respected jobs.
❖ 排在底層：是境況最差的人—那些錢最少、教育程度最低

10	•
9	•
8	•
7	•
6	•

、工作最不受尊敬或沒有工作的人。<br>At the bottom are the people who are the worst off – those who have the least money, least education, the least respected jobs, orno job.<br>❖ 在這個梯子上越高，您就越接近頂層的人；越低，就表示您越接近底層的人。<br>The higher up you are on this ladder, the closer you are to the people at the very top; the lower you are, the closer you are to the people at the very bottom.<br><br>**您會把自己放在梯子的哪個位置呢？**<br>**Where would you place yourself on this ladder?**<br>請從1-10處標出您在臺灣認爲自己目前相對於其他人在您生命中的位置。<br>Please mark a field from 1-10 where you think you stand at this time in your life relative to other people in TAIWAN.	5<br>4<br>3<br>2<br>1	•<br>•<br>•<br>•<br>•

11. **您主要如何支付您的學費與生活費？（可複選）**
How do you primarily finance your studies? (You can select multiple response options if necessary.)

- 由父母支付
  Support by parents
- 學生津貼（例如：助理費、獎助學金、其他單位的固定資助等）
  Student grant, Scholarship
- 在學期中工作（有正職工作）
  Employment during the semester
- 在學期當中的休息時間打工（非正職工作）
  Employment during the semester break
- 就學貸款
  School loan
- 其他，請填寫_______________
  Other, namely _______________

以下是詢問您目前生活狀況和感到負擔的程度。（共2題）

The following is an assessment of your current life situation and the extent to which you feel burdened by it.

12. 您如何看待目前的生活狀況？

How do you personally find your current life situation in general?

可駕馭的<br>Manageable	•	•	•	•	•	•	•	不可駕馭的<br>Unmanageable
沒意義的<br>Meaningless	•	•	•	•	•	•	•	有意義的<br>Meaningful
有條理的<br>Structured	•	•	•	•	•	•	•	紊亂的<br>unstructured
易受影響的<br>Easy to influence	•	•	•	•	•	•	•	沒有受到影響的<br>Impossible to influence
微不足道的<br>Insignificant	•	•	•	•	•	•	•	明顯重要的<br>Significant
清楚的<br>Clear	•	•	•	•	•	•	•	不清楚的<br>Unclear
可控制的<br>Controllable	•	•	•	•	•	•	•	不可控制的<br>Uncontrollable
沒有回報的<br>Unrewarding	•	•	•	•	•	•	•	有回報的<br>Rewarding
可預見的<br>Predictable	•	•	•	•	•	•	•	不可預見的<br>Unpredictable

13. 以下的聲明關乎您對未來的態度。每句話都可以不同程度地反映您的態度。（如果某句話準確地描述了您的態度，就應答爲「絕對正確」； 如該陳述不能準確描述您的態度，請應答爲「絕對錯誤」）

The statements below concern your attitude towards the future. Each statement can reflect your attitude to different degrees.

(If a certain statement describes your attitude exactly, answer it with "decidedly true". If the statement is not an accurate description of your attitude, answer it with "decidedly false".)

	絕對錯誤<br>Decidedly false			很難說<br>Hard to say			絕對正確<br>Decidedly true
我擔心現在困擾我的問題會持續很久。<br>I am afraid that the problems which trouble me now will continue for a long time	•	•	•	•	•	•	•
我一想到自己有時可能面臨生活的危機或困難，就感到害怕。<br>I am terrified by the thought that I might some-times face life's crises or difficulties	•	•	•	•	•	•	•
我擔心將來我的生活會越來越糟。<br>I am afraid that in the future my life will change	•	•	•	•	•	•	•

for the worse							
我擔心經濟和政治形勢的變化會威脅我的未來。<br>I am afraid that changes in the economic and political situation will threaten my future	•	•	•	•	•	•	•
想到將來我無法實現我的目標，我感到不安。<br>I am disturbed by the thought that in the future I will not be able to realize my goals	•	•	•	•	•	•	•
我一想到自己未來的事，就感到緊張不安。<br>I fall into a state of tension and uneasiness when I think of my future affairs	•	•	•	•	•	•	•
我確信將來我會實現我一生中最重要的目標（價值）。<br>I am sure that in the future I will realize the most important goals (values) in my life	•	•	•	•	•	•	•
我覺得世界快要崩潰了。<br>I have the impression that the world tends toward collapse	•	•	•	•	•	•	•
我擔心我可能會突然發生意外或得到嚴重疾病（例如：癌症、COVID-19等)<br>I am disturbed by the possibility of a sudden accident or serious illness (e.g. cancer, COVID-19)	•	•	•	•	•	•	•

下列的問題是有關你處理冠狀病毒資訊有多容易或困難。（共6題）

Following questions are about how easy or difficult it is for you to deal with information about the corona-virus.

14. 您在過去的四個禮拜裡，有搜尋過冠狀病毒的資訊嗎？（這包括感染個案、避免或處理日常生活限制等資料）

Have you searched the Internet in the last four weeks for information about the coronavirus? (This may include, for example, information on infected cases, on avoiding or dealing with restrictions in everyday life.)

- 是，只有找提供自己參考的資料。
  Yes, only information for me
- 是，只有找給其他人的資料。
  Yes, only information for other people
- 是，有找資料給自己，同時提供給他人的資料。
  Yes, information for me and other people
- 沒有，我最近四個禮拜沒有查找任何資料（請直接跳到第**24**題）。
  No, I haven't searched for information in the last four weeks(➲ please proceed with question 24).

15. 對於在網路上搜尋冠狀病毒及相關主題，對您而言是否容易？

When you search the Internet for information on the coronavirus or related topics, how easyor difficult is it for you to…

	非常簡單 Very easy	簡單 Easy	困難 Difficult	非常困難 Very difficult
… 從您所找到的資料中作出選擇？ make a choice from all the information you find?	•	•	•	•
… 使用適當的字句或搜尋疑問來找到您要所尋找的資料？ use the proper words or search query to find the information you are looking for?	•	•	•	•
… 找到您要找的確切資料？ find the exact information you are looking for?	•	•	•	•

16. 在論壇、Facebook、或推特等社群媒體輸入有關冠狀病毒及相關主題的訊息時，請回答下列狀況對您而言的難易程度…

When typing a message (e.g. on a forum, or on social media such as Facebook or Twitter) about the coronavirus or related topics, how easy or difficult is it for you to…

	非常簡單 Very easy	簡單 Easy	困難 Difficult	非常困難 Very difficult
… 清楚表達您的問題或與健康有關的憂慮？ clearly formulate your question or health-related worry?	•	•	•	•
… 用書面表達您的意見、想法或感情？ express your opinion, thoughts, or feelings in writing?	•	•	•	•
… 把訊息寫成怎樣,讓別人明白您的意思？ write your message as such, for people to understand exactly what you mean?	•	•	•	•

17. 當您在網上搜索冠狀病毒或相關話題時，請回答下列狀況對您而言的難易程度⋯

When you search the Internet for information on the coronavirus or related topics, how easy or difficult is it for you to…

	非常簡單 Very easy	簡單 Easy	困難 Difficult	非常困難 Very difficult
… 判斷相關的資料是否可靠？<br>decide whether the information is reliable or not?	•	•	•	•
… 判斷資料是否與商業利益有關（例如：由試圖出售產品的人所撰寫的訊息）？<br>decide whether the information is written with commercial interests (e.g. by people trying to sell a product)?	•	•	•	•
⋯ 查閱不同的網站，以查看它們是否提供相同的資料？<br>check different websites to see whether they provide the same information?	•	•	•	•
… 決定您所找到的資料是否適合您？<br>decide if the information you found is applicable to you?	•	•	•	•
… 應用您在日常生活中發現的狀況？<br>apply the information you found in your daily life?	•	•	•	•
⋯ 使用您所找到的資料來決定您的健康（例如有關保護措施、衛生規例、傳播途徑、風險及預防方法等）？<br>Use the information you found to make decisions about your health (e.g. on protective measures, hygiene regulations, transmission routes, risks and their prevention)?	•	•	•	•

18. 當您在公共論壇或社交媒體上發佈有關冠狀病毒或相關主題的訊息時，您有多常會發生下列的情況⋯

When you post a message about the coronavirus or related topics on a public forum or social media, how often…

	未曾 Never	一次 Once	幾次 Several time	常常 Often
… 您覺得很難判斷誰會閱讀到？<br>do you find it difficult to judge who can read along?	•	•	•	•
… 您是否（偶然或無意）分享您的個人資料（例如：姓名或地址）？<br>do you (intentionally or unintentionally) share your own private information (e.g. name or address)?	•	•	•	•
… 您（偶然或無意）分享他人的個人資料嗎？<br>do you (intentionally or unintentionally) share some else’s private information?	•	•	•	•

下面是針對冠狀病毒在臺灣和全世界傳播的現狀。下列問題是指搜尋與冠狀病毒有關的資訊，以及爲您個人生活帶來的所有影響。（共 5 題）

In the following, the current situation regarding the spread of the coronavirus in Taiwan and worldwide is addressed. The following questions refer to the search for information related to the coronavirus and all consequences for your personal life.

19. 現在，在網絡上出現了各種獲取冠狀病毒相關資訊的可能性。 請註明您多常使用這些資料。

Now various possibilities are mentioned how to get information about the coronavirus and related topics on the Internet. Please indicate how often you currently use these sources.

	常常 Often	有時 Sometimes	不常 Rarely	不曾 Never	不知道 Don´t know
搜尋引擎（例如：Google、Yahoo等) Search engines (e.g. Google, Yahoo!)	•	•	•	•	•
公營機構網站（例如：衛福部、各縣市相關網站、或是在臺灣具有公股性質的公司也包括，像是hinet等） Websites of public bodies	•	•	•	•	•
維基百科和其他在線百科全書 Wikipedia and other online-encyclopaedias	•	•	•	•	•
社群媒體（例如：Facebook、IG、Twitter等） Social media (e.g. Facebook, Instagram, Twitter)	•	•	•	•	•
YouTube	•	•	•	•	•
健康專題博客 Blogs on health topics	•	•	•	•	•
社區指南（例如：在臺灣有關社區相關的網站、里長或地區衛生所相關網站均包括在內) Guidebook-communities	•	•	•	•	•
健康門戶網站（例如：以健康為訴求的臺灣網站均包括在內） Health portals	•	•	•	•	•
醫師個人或健康保險公司等相關的網站 Websites of doctors or health insurance companies	•	•	•	•	•
新聞入口網站（例如：臺灣的報紙、電視台等入口網站） News portals (e.g. of newspapers, TV stations)	•	•	•	•	•

20. 您都使用什麼語言來搜尋冠狀病毒及相關健康資訊？可複選。

What language do the sources have that you use for searching information on coronavirus and related health topics? (You can select multiple response options if necessary.)

- 中文 Chinese
- 英文 English
- 其他語言（不包括中文或英文）Other languages than Chinese or English, namely: ______

21. 您會搜尋下列哪些有關關狀病毒相關的主題？可複選。

Please indicate the specific topics you are searching for in the context of the coronavirus.
(You can select multiple response options if necessary.)

- 當前冠狀病毒傳播的情況（例如：受感染個案數目）
  Current spread of the coronavirus (e.g. number of infected cases)
- 冠狀病毒傳播途徑
  Transmission routes of the coronavirus
- COVID-19的症狀
  Symptoms of COVID-19
- 個別預防感染措施（例如：洗手、酒精消毒等）
  Individual measures to protect against infection (e.g. hand washing tips)
- 衛生規例（例如：社區消毒或清潔等）
  Hygiene regulations (e.g. disinfection & cleaning)
- 時局評估及建議（例如：臺灣的衛福部等相關網站均包括）
  Current situation assessments and recommendations (e.g. Taiwan's Ministry of Health and Welfare and other related websites)
- 限制（例如：出境限制、居家令）
  Restrictions (e.g. exit restrictions, stay-at-home orders)
- 冠狀病毒的經濟和社會後果
  Economic and social consequences of the coronavirus
- 處理冠狀病毒引起的心理壓力
  Dealing with psychological stress caused by the coronavirus
- 其他。請填寫________________________________
  Others, namely: ________________________________

22. 下列問題希望了解當您上網搜索冠狀病毒及相關話題資訊時，下列各種狀況對您的重要程度？

Now it's about how important various things are to you when you search the Internet for coronavirus and related topics. How important is it to you that...

	非常重要<br>Very important	比較重要<br>Rather important	比較不重要<br>Rather not important	不重要<br>Not at all important
… 資料是否已經更新？<br>the information is up to date?	•	•	•	•
… 資料是否已經證實？<br>the information is verified?	•	•	•	•
… 您很快瞭解了您最重視的訊息？<br>you quickly learn the most important things?	•	•	•	•
… 資訊來自官方嗎？<br>the information comes from official sources?	•	•	•	•
… 不同意見的出現？<br>different opinions are represented?	•	•	•	•
… 這課題是否已全面處理？<br>the subject is dealt with comprehensively?	•	•	•	•

23. 您對於在網上所找到的冠狀病毒資訊滿意嗎？
How satisfied are you with the information you find on the Internet about coronavirus?

- 非常不滿意 Very dissatisfied
- 不滿意 Dissatisfied
- 普通 Partly satisfied
- 滿意 Satisfied
- 非常滿意 Very satisfied

24 . 對COVID-19的恐懼

Fear of COVID-19

	請填選您與下列敘述的相符程度<br>Please let us know your level of agreement with the statements below	強烈不同意<br>Strongly disagree	不同意<br>Disagree	中立<br>Neither agree nor disagree	同意<br>Agree	強烈同意<br>Strongly agree
1.1	我非常害怕 COVID-19。<br>I am most afraid of COVID-19.	1☐	2☐	3☐	4☐	5☐
1.2	想到 COVID-19 會讓我很不舒服。<br>It makes me uncomfortable to think about COVID-19.	1☐	2☐	3☐	4☐	5☐
1.3	當我想到 COVID-19 我的手會發冷。<br>My hands become clammy when I think about COVID-19.	1☐	2☐	3☐	4☐	5☐
1.4	我怕我會因為 COVID-19 而死亡。<br>I am afraid of losing my life because of COVID-19.	1☐	2☐	3☐	4☐	5☐
1.5	當我在社群軟體裡看 COVID-19 的新聞和故事時，我會感到緊張或焦慮。<br>When I watch news and stories about COVID-19 on social media, I become nervous or anxious.	1☐	2☐	3☐	4☐	5☐
1.6	我會因為擔心得到 COVID-19 而失眠。<br>I cannot sleep because I'm worrying about getting COVID-19.	1☐	2☐	3☐	4☐	5☐
1.7	當我想到 COVID-19 時，我會心跳加速和心悸。<br>My heart races or palpitates when I think about getting COVID-19.	1☐	2☐	3☐	4☐	5☐

完成了！非常感謝您的細心填答，感恩！

You made it! Thank you very much for your participation.
